# Standardised and Objective Dietary Intake Assessment Tool (SODIAT): Protocol of a dual-site dietary intervention study to integrate dietary assessment methods

**DOI:** 10.12688/f1000research.155683.1

**Published:** 2024-10-07

**Authors:** Eka Bobokhidze, Michelle Weech, Katerina Petropoulou, Thomas Wilson, Jennifer Pugh, Rosalind Fallaize, Isabel Garcia-Perez, Frank P.-W. Lo, Adrian R Solis, Juliet Vickar, Stamatia Giannarou, George Mylonas, Benny Lo, Amanda J Lloyd, Albert Koulman, Manfred Beckmann, John Draper, Gary Frost, Julie A Lovegrove

**Affiliations:** 1 Hugh Sinclair Unit of Human Nutrition and Institute for Cardiovascular and Metabolic Research, University of Reading, Reading, England, RG6 6DZ, UK; 2 Section of Nutrition, Department of Metabolism, Digestion and Reproduction, Imperial College London, London, England, W12 0NN, UK; 3 Department of Life Sciences, Aberystwyth University, Aberystwyth, Wales, SY23 3DA, UK; 4 School of Life and Medical Sciences, University of Hertfordshire, Hatfield, England, AL 10 9AB, UK; 5 The Hamlyn Centre for Robotic Surgery, Imperial College London, London, England, SW7 2AZ, UK; 6 Nutritional Biomarker Laboratory, MRC Epidemiology Unit, University of Cambridge, Cambridge, England, CB2 0QQ, UK

**Keywords:** Nutrition, Health, Research, Dietary reporting, Underreporting, Misreporting, Biomarkers

## Abstract

**Introduction:**

Current dietary assessment methods face challenges in accurately capturing individuals’ dietary habits, undermining the efficacy of public health strategies. The ‘Standardised and Objective Dietary Intake Assessment Tool’ (SODIAT)-1 study aims to assess the effectiveness of three emerging technologies (urine and capillary blood biomarkers, and wearable camera technology) and two online self-reporting dietary assessment tools to monitor dietary intake.

**Methods:**

This randomised controlled crossover trial will recruit 30 participants (aged 18-70 years and BMI of 20-30 kg/m ^2^) from Imperial College London and the University of Reading. Exclusion criteria include recent weight change, food allergies/intolerances, following restrictive diets, certain health conditions and medication use. Interested volunteers will be directed to an online screening questionnaire via REDCap and eligible participants will attend a pre-study visit. Volunteers will consume, in a random order, two highly-controlled diets (compliant and non-compliant with UK guidelines) for four days each. Each study arm will be separated by at least one-week. During each test period, dietary intake will be monitored continuously using wearable cameras and self-recorded using eNutri (food frequency questionnaire) and Intake24 (24-hour dietary recall). Urine and capillary blood samples will be collected for biomarker analysis. Data analysis will assess the accuracy of dietary reporting across these methods using Lin’s concordance correlation coefficient.

**Discussion and ethical considerations:**

This study introduces a novel approach to dietary assessment, addressing the limitations of traditional methods by reducing misreporting and enhancing inclusivity, particularly for underrepresented populations with literacy or language barriers. However, challenges persist, such as variability in biomarker data due to failure to adhere to sample storage requirements and the practicalities of continuously wearing cameras. To protect privacy, participants will be instructed to remove cameras at inappropriate times, and artificial intelligence will be used to blur all images captured apart from food.

## Introduction

Optimal nutrition is fundamental for maintaining good health and preventing diseases across the life course. ^1^ The relationship between dietary habits and noncommunicable diseases (NCDs) is globally recognised, ^2, 3^ and evidence suggests unhealthy diets are strongly associated with increased prevalence of diabetes, various forms of cancer, and cardiovascular diseases. ^4– 6^ The impact of diet on population health has been highlighted by the Global Burden on Disease study that reported over 11 million deaths worldwide per year can be attributed to suboptimal diets. ^7^

Nutrition research has a significant role in reducing the detrimental health impact of poor diets globally. Assessing population food intake can provide substantial data on the nation’s nutritional status and may be used in planning and implementation of evidence-based public health interventions. ^8^ However, lack of accurate dietary assessment measures continuously undermines the strength and efficacy of public health strategies. ^9, 10^ Traditional subjective methods of dietary assessment, such as food frequency questionnaires (FFQs), 24-hour dietary recalls, and food diaries, are widely used to capture individuals’ dietary information. ^11^ Despite being non-invasive, easy to use and suitable for large scale studies (depending on method), these self-reporting methods face notable challenges that can compromise the accuracy and reliability of collected dietary data. ^12– 15^ Memory recall bias is a common challenge, as participants may struggle to remember all consumed items, leading to potential underreporting or inaccuracies, ^14^ whereas social desirability bias occurs when respondents align their reported food choices with perceived societal expectations, impacting the representation of true dietary habits. ^16^ In addition, estimating portion sizes poses a challenge, as individuals may struggle with accuracy and perceptions of portion sizes differ between individuals (e.g., small, medium and large). ^17^ Finally, response burden and fatigue, particularly prevalent when individuals weigh and record their dietary consumption over extended periods, can result in incomplete records, whereas day-to-day variability in dietary habits (such as oily fish and alcohol that are not typically eaten daily) may not be adequately captured for shorter recording periods. ^9^

Cultural and social influences, lack of standardisation in reporting procedures, and limited detail on food preparation methods further contribute to the complexity of self-reported dietary assessments. ^13^ The phenomenon of underreporting or overreporting in dietary assessments adds another layer of complexity, for example, obese individuals, particularly women, are most likely to underestimate their energy intake by under-reporting high energy foods considered socially undesirable. ^18^ Mitigating these challenges and improving accuracy is essential for advancing the assessment methodology of population food intake. ^14^

In addition to subjective measures, objective methods can also be used to assess dietary intake. These do not rely on participants’ self-reported intakes, instead dietary intake is assessed using various physical, biochemical, physiological or environmental measures, such as direct observation, nutritional biomarkers and duplicate diets. ^12^ The development of advanced technologies, such as sensor-based and image-based tools, has increased the possibilities to address the limitations of self-reporting in nutrition research. ^19^ Additionally, detecting dietary biomarkers in bodily fluids can reflect food intake and complement traditional dietary assessment methods. ^20^ Implementing these methods minimise the biases associated with subjective measures, however, each objective method has its own limitations, and there is no universally accepted “gold standard”. ^12^

To address this problem, the ‘Standardised and Objective Dietary Intake Assessment Tool’ (‘ SODIAT’)-1 study will explore the ability of three emerging objective technologies and two subjective online tools to accurately assess what people eat and drink. The objective measures will include: 1. Urine biomarker metabolomics, ^21^ 2. Capillary blood biomarkers ^22^ and 3. Wearable camera technology, ^23^ and the subjective measures will include eNutri (online FFQ tool) ^24^ and Intake24 (online 24h recall tool). ^13, 24, 25^ The primary objective of the study is to calibrate the above dietary assessment technologies and tools for effectiveness to monitor exposure to foods/food groups commonly consumed in the UK in a controlled diet intervention. By conducting a comprehensive evaluation of their capabilities in accurately reporting dietary intake, the study aims to identify the most promising features of each technology. Subsequently, the research team will collaboratively integrate these features to create a combined optimal tool that maximises accuracy and usability which will be tested in future studies in the home environment (not described in this protocol).

## Methods

### Study population

In randomised cross over study SoDiat-1 thirty male and female participants aged between 18-70 years will be recruited by the research teams at Imperial College London and University of Reading, with an equal distribution per study location. Participants of all ethnicities with a body mass index (BMI) 20-30 kg/m ^2^ will be eligible. Exclusion criteria are as follows:

• Involvement in any other study during the previous 12 weeks, is unable to commit to the study (e.g., travel commitments) or unwilling to collect urine and blood samples and wear the micro-camera.
• A weight change of more than 3kg in the preceding 3 months or following a weight-loss diet.
• Excess alcohol intake (more than 21 alcohol units per week).
• Unwilling to abstain from drinking alcohol and avoid strenuous exercise during the two 5-day test periods.
• Unwilling to follow the study menus (e.g., dislike of food items, following a restrictive/specialised diet or receiving specialised dietary advice for a medical condition).
• Unable to eat fish and/or meat (e.g., are vegan or vegetarian).
• Allergy/intolerance to any of the food items in the menu.
• Use of dietary supplements (e.g., multivitamins, fish oils), unless willing to have a washout of at least 2 weeks prior to taking part in the study.
• Pregnant or lactating.
• Diagnosed with any of the following: eating disorder, diabetes, cancer, gastrointestinal disorders (e.g., inflammatory bowel disease or irritable bowel syndrome), kidney disease, liver disease, pancreatitis, HIV or AIDS or any other chronic illness.
• Taking any of the following medications: anti-inflammatory drugs or steroids, antibiotics, androgens, phenytoin, erythromycin, or thyroid hormones.
• Illicit substance use.
• Diagnosed with dementia or other conditions affecting memory.
• Difficulty using laptops/tablets (e.g., cannot use these devices without assistance, are blind or have other conditions affecting sight, or have physical disabilities/conditions that affect ability to press buttons).
• Cannot read and understand English.

### Recruitment

Various methods of recruitment will be employed, including distributing posters around the university campuses, emails sent to the respective clinical unit’s volunteer databases and university mailing lists, and social media advertisements for groups within the universities and surrounding areas. Recruitment will start in December 2023 and finish when required sample size is achieved. Recruitment materials will include a link and QR code that takes interested participants to REDCap, a secure web application for administering online surveys and recording datasets in research studies ( https://www.project-redcap.org/), where they can view and download the participant information sheet.

### Study design

Screening

Interested participants will complete an online screening questionnaire on REDCap, after which the respective research teams will determine their eligibility and/or request further information from the interested volunteers.

Consent and pre-study visit

**Figure 1.**
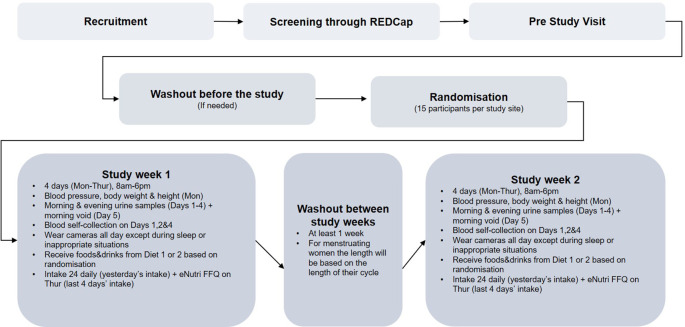
A schematic diagram of SoDiat Study-1.

Prior to starting the study, participants will attend the NIHR Imperial Clinical Research Facility at Hammersmith Hospital or the Hugh Sinclair Unit of Human Nutrition (HSUHN) at the University of Reading (depending on their preferred location as specified on the screening form). Researchers will explain the study in full, reconfirm eligibility (such as ensuring all food items on the menu can be consumed) and allow the participant to ask questions before informed written consent will be taken. During the pre-study visits, participants will be provided with a study handbook and the technologies used during the study will be explained and demonstrated to them. Their self-reported BMI will also be confirmed by measuring height and weight using a bioelectrical impedance analyser (Tanita MC780 MA P (Imperial) and BC-418 (Reading), TANITA UK Ltd, UK) to ensure the participant is within the correct BMI range. If the volunteer is happy to proceed, they will be invited to schedule the two 4-day study visits. They will also be provided with urine kits to take home and a reminder checklist/log form for the evening/morning prior to their first visit. A schematic diagram ( Figure 1) illustrating the study process from recruitment through to completion.

### Randomisation

After the pre-study visit, participants will be assigned a study ID code and randomisation will be undertaken using REDCap. Participants will be randomly allocated to one of two diet orders: Diet 1 followed by Diet 2 or Diet 2 followed by Diet 1. Randomisation will be stratified by study centres. The research team and participant will not be blinded to the randomisation, as study menus are provided in advance, making it clear which diet they will follow each week.

### Study diets

Participants will consume two controlled diets, one per study period, provided in a random order: Diet 1: non-compliant with UK dietary guidelines (e.g., high in saturated fat, free sugars, and salt and low in fibre); Diet 2: compliant with UK dietary guidelines, e.g., within the dietary reference values for saturated fat (≤10% total energy (TE)), free sugars (≤5 %TE), salt (≤6 g/d) and fibre (≥30 g/d) ( Table 1). The diets are matched for energy, protein, total fat and carbohydrate. Foods and drinks selected for each diet were selected to allow investigation of specific biomarkers. ^21^ Each diet consists of a 2-day repeating menu (e.g., menu A served on days 1 and 3, and menu B served on days 2 and 4) as shown in Tables 2 and 3. Bottled spring water will also be available to drink throughout the day. Meals and snacks will be consumed at 2-hour intervals throughout the study days (9 am breakfast, 11 am morning snack, 1 pm lunch, 3 pm afternoon snack, and 5 pm dinner) and will be served using identical tableware at each research unit (white crockery and clear glass on days 1 and 2 and patterned crockery and coloured glass on days 3 and 4); no foods/drinks will be consumed directly from their packaging. Participants will also be provided with a snack and bottled water to take home to consume before 8 pm and instructed not to eat or drink anything else before returning to the research unit the following day.

**Table 1.** Nutrient composition of the study diets *.

Nutrient	Diet A	Diet B
Energy (kcal/d)	2291	2168
Protein (%TE)	14.8	14.3
Total fat (%TE)	35.6	35.6
Saturated fat (%TE)	16.4	5.7
Carbohydrates (%TE)	46.8	43.5
Free sugars (%TE)	13.6	4.6
Dietary fibre (AOAC) (g/d)	21.8	38.8
Salt (g/d)	7.6	5.6

* Mean of menu 1 and menu 2 per study diet.

**Table 2.** Study menus for diet 1.

Menu	Menu items and quantities
**Menu A**	
**Breakfast**	Pain au chocolat (chocolate filled pastry) (45g)
	Honey nut cornflakes (30g) with whole milk (200g)
	Tea (245g) with whole milk (25g) and sugar (5g)
**Morning snack**	Cheese-flavoured crackers (23g)
	Instant coffee (230g) with whole milk (40g) and sugar (5g)
**Lunch**	Pepperoni, ham and mushroom pizza baguettes (250g)
**Afternoon snack**	Chocolate-coated caramel wafer bar (30g)
	Chocolate-flavoured milkshake mix (20g) with whole milk (250g)
**Dinner**	Beef lasagne ready meal (400g) with frozen mixed vegetables (carrots, peas and green beans) (68g)
	Apple and raspberry juice drink (250g)
**Evening snack**	Salt and vinegar potato crisps (25g)
**Menu B**	
**Breakfast**	White bread toasted (80g) with spreadable butter (20g)
	Baked beans and pork sausages canned in tomato sauce (208g)
	Tea (245g) with whole milk (25g) and sugar (5g)
**Morning snack**	Coconut macaroon (chewy coconut ‘biscuit’) (30g)
	Instant coffee (230g) with whole milk (40g) (no sugar)
**Lunch**	Chicken and bacon pasta in a creamy sauce ready meal (400g)
**Afternoon snack**	Ready salted potato crisps (18g)
	Fizzy orange drink (330g)
**Dinner**	Beef stroganoff in a creamy mushroom sauce with rice ready meal (400g) with frozen mixed vegetables (carrots, broccoli and sweetcorn) (68g)
	Apple juice (200g)
**Evening snack**	Chocolate covered wafer biscuit bar (32g)

**Table 3.** Study menus for diet 2.

Menu	Menu items and quantities
**Menu A**	
**Breakfast**	Muesli with dried fruits (50g) and unsweetened soya drink (200g)
	Wholemeal seeded bread toasted (50g) with peanut butter (25g)
	Tea (240g) with skimmed milk (30g)
**Morning snack**	Dried apple rings (30g)
	0% fat Greek style flavoured yogurt (115g)
	Instant coffee (220g) with skimmed milk (50g)
**Lunch**	Chicken and pasta in a spicy tomato sauce ready meal (400g) with frozen mixed vegetables (carrots, broccoli and sweetcorn (68g)
	Mandarins in juice (113g)
**Afternoon snack**	Reduced salt green pitted olives (30g), cherry tomatoes (75g) and breadsticks (20g)
**Dinner**	Vegetable Biryani (mildly spiced mixed roasted vegetables with basmati rice) ready meal (400g)
	Onion bhaji (50g)
**Evening snack**	Reduced salt potato crisps (25g)
**Menu B**	
**Breakfast**	Weetabix (38g) with hazelnuts (15g), frozen blueberries (80g) and oat drink (120g)
	Wholemeal bread toasted (44g) with sunflower spread (10g)
	Tea (240g) with skimmed milk (30g)
**Morning snack**	Peaches in juice (113g) & whole almonds (30g)
	Coffee (220g) with skimmed milk (50g)
**Lunch**	Moroccan spiced chicken and chickpea soup (300g) with wholemeal bread roll (54g) and sunflower spread (15g) Red grapes (80g)
**Afternoon snack**	Houmous dip (50g) with lightly salted tortilla chips (30g) Sugar-free lemonade (330g)
**Dinner**	Salmon pie (salmon in a cream sauce topped with mashed potato and cheese) ready meal (375g) with frozen mixed vegetables (carrots, peas and green beans) (68g)
	Fruit flavoured water drink with sweeteners (250g)
**Evening snack**	Dried fruit and nut chewy bar (30g)

### Study visits

The study visits will take place at the NIHR/Imperial Clinical Research Facility at Hammersmith Hospital or the HSUHN at the University of Reading and will include two study periods each consisting of four full-day (8 am to 6 pm) visits from Monday to Thursday with a short visit on the fifth study day (Friday) to return final urine samples and all study equipment. A washout period of at least one week between study periods will be required (menstruating women will attend study visits at the same phase of their menstrual cycle).

### Study visit procedures

The day before starting each study period, participants will be asked to restrict their caffeine and alcohol intakes and exercise levels to amounts that are usual for them and fast for 12 hours overnight (not consuming any food or drink, except water). Upon waking, participants will also collect a first morning void (FMV) urine sample.

Participants will attend the research unit at 8 am on day 1 (Monday) of each study period. Upon arrival, blood pressure, height and body weight will be measured. A fasted capillary blood sample (OneDraw) will also be self-collected and participants will be set up with the wearable camera (which will be worn continuously) before being provided with breakfast. Habitual diet will be recorded using the eNutri tool. With the exception of mealtimes, participants will have the rest of the day as free time but must remain in the research unit. At the end of the study day (6 pm), participants will be provided with bottled water and a snack for the evening, urine kits (for last evening void (LEV) and FMV samples) and a reminder checklist/log. Days 2-4 will repeat day 1 except: 1) Intake24 will be used on days 2-4 to record dietary intake during the previous 24 hours, 2) eNutri will be repeated at the end of day 4 (Thursday) to record dietary intake during the previous 4 days, and 3) capillary blood samples will not be collected on day 3 (Wednesday). On day 5 (Friday), participants will collect a final FMV, complete Intake24 and return samples/equipment to the research unit.

### Objective dietary assessment tools

Spot urine samples

**Figure 2.**
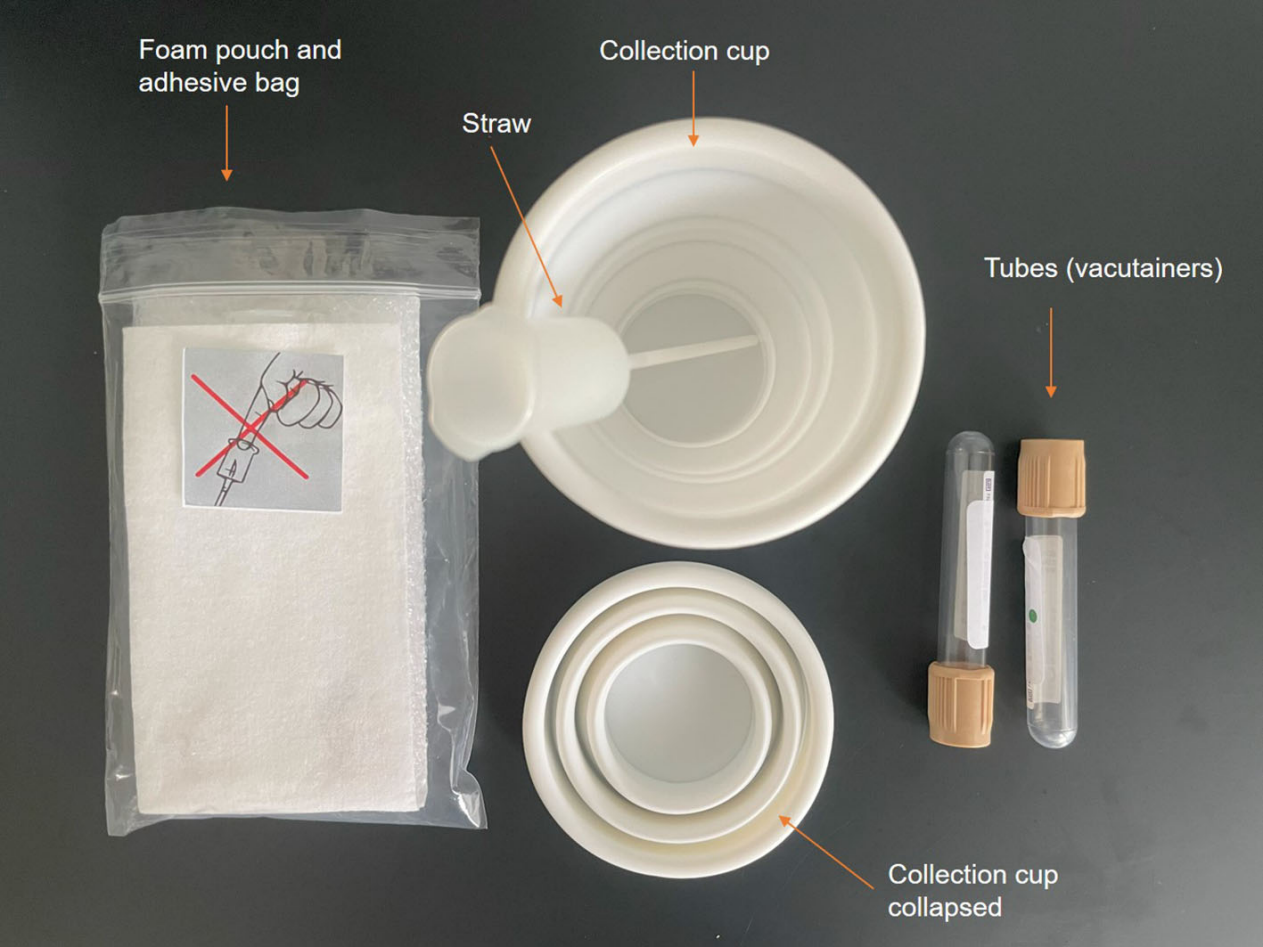
Urine transfer and straw kit.

Participants will collect spot urine samples using previously described methods. ^21^ For each collection, participants will be provided with four additive-free vacuum collection tubes (4 mL) (plus two spares), urine transfer straws and a disposable collection cup ( Figure 2). Participants will collect their FMV urine and LEV urine for all four study days in each study period as well as a FMV sample on day 5 using the collection cup. Participants will then transfer samples to four tubes via the transfer straw and store at 4 °C. During each study day, samples will be processed in the research unit using previously described methods to render them acellular then they will be stored at -80 °C until the end of the study. ^26^

Urinary biomarkers of dietary intake will be measured at Aberystwyth University using a combination of Ultra-High Performance Liquid Chromatography (UHPLC) Triple Quadrupole Mass Spectrometry (QqQ-MS) and high-resolution mass spectrometry (HRMS). Previous studies have determined a list of dietary intake biomarkers that reflect intake of common UK diet components and are sufficiently robust and reproducible in spot urine samples from dietary intervention studies. ^27^ Global dietary patterns will be assessed by measuring the urine samples using Proton Nuclear Magnetic Resonance ( ^1^H-NMR) at Imperial College London. A global dietary score will be generated from the 1H-NMR urinary metabolic profiles following a previously validated methodology ^28^ that will indicate the quality of the diet in combination with a complementary set of urinary dietary biomarkers.

Capillary blood samples

**Figure 3.**
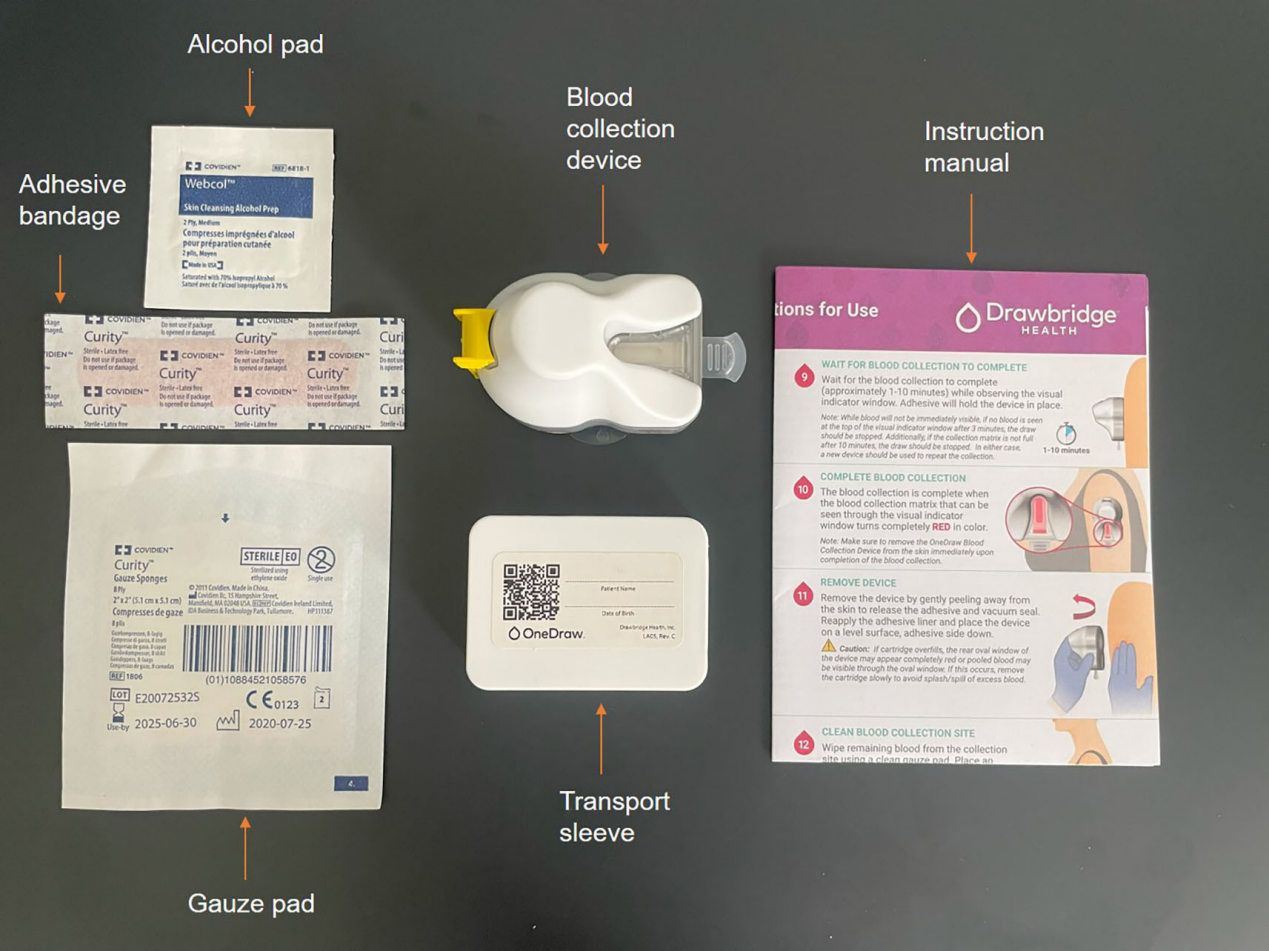
OneDraw blood collection kit.

Capillary blood samples will be self-collected by participants prior to breakfast on days 1, 2 and 4 using a OneDraw kit (Drawbridge, Thorne Research, Summerville SC, US) as shown in Figure 3. The single-use device attaches to the upper arm or thigh via a hydrogel adhesive and vacuum and collects capillary blood with little discomfort for the participant. The capillary blood is directly collected onto two paper strips. ^22^ When the collection is finished, the cartridge containing the blood samples will be placed in the transport sleeve then left at room temperature for at least 48 hours to allow the blood to dry prior to storage at -80 °C.

The dried blood samples will be extracted at the University of Cambridge using a standard protocol for dried blood spots ^29^ and the lipid profile will be analysed using a combination of UHPLC and HRMS, and lipids will be quantified against internal standards as published previously. ^22^

Wearable camera technology

To effectively capture the dietary habits and food-related activities of individuals in UK households, a comprehensive passive dietary assessment system has been meticulously designed for this study. This system is a fusion of both hardware and software components, each with distinct functionalities to enhance the accuracy and efficiency of dietary data collection.

Hardware

**Figure 4.**
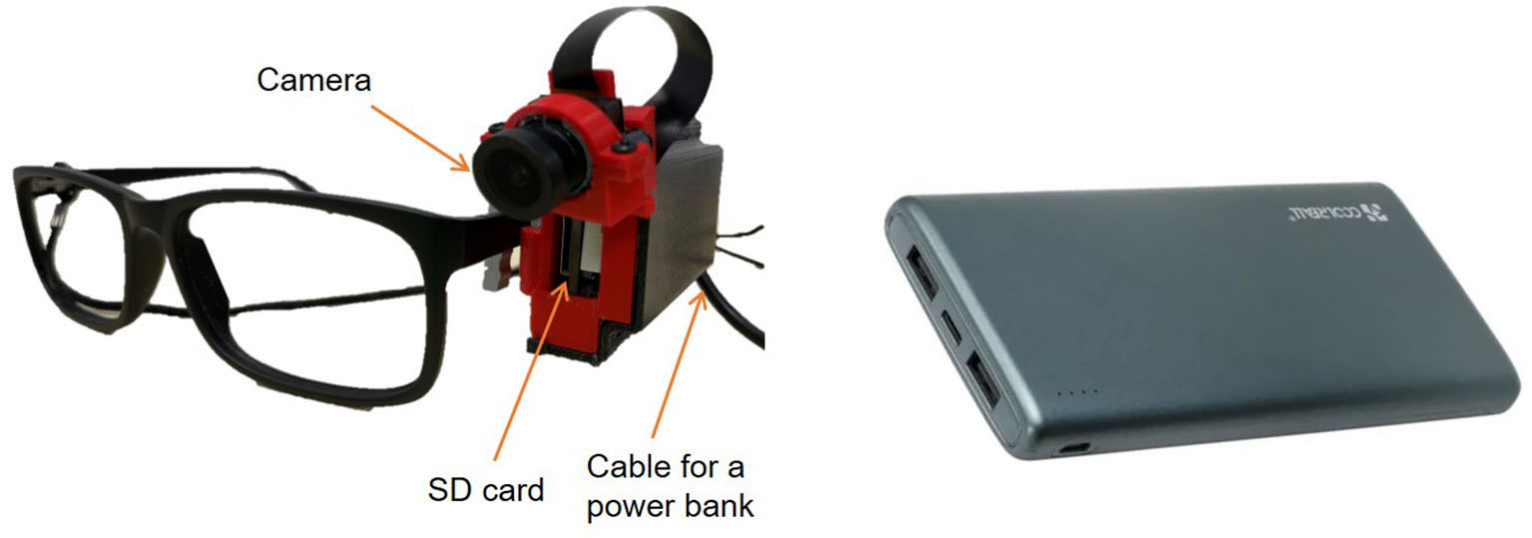
Wearable camera equipment.

For this pilot study, prototype of the camera was developed - wearable camera capable of recording up to 20hrs called M2.1. The wearable camera device is a high-definition camera with a maximum resolution of 2592x1944 pixels, mounted on the side arm of lens-less eye glasses frames to align with the user’s viewpoint and is connected to a rechargeable powerpack whilst in use ( Figure 4). It is designed to be used during daytime, capturing images of the eating process at frequency of one image every 1.5 seconds. The camera is powered by a built-in STM32 microcontroller with a 32-bit arm processor. The device begins recording upon the insertion of an empty 128 GB SD card and the camera is turned on. The camera is turned on automatically once is connected to the external battery.

Prior to breakfast on day 1, participants will receive a camera device mounted on a glasses frame (or they can mount this on their own glasses frame) and instructed to wear this continuously until they go to bed, with the exception of when it is not suitable for the camera to be worn (such as when getting dressed and using the bathroom) in which case the glasses will be temporarily removed and details noted on the camera log. Upon arrival to the research unit on days 2-5, the SD cards will be removed from the camera device by the research team and uploaded in duplicate to two encrypted external hard drives. Whilst the cameras are not in use overnight, participants will be instructed to fully charge the power packs and start wearing the cameras the following morning before returning to the research unit (days 2-4).

Software

To ensure the anonymity of individuals, all footage captured by wearable cameras will undergo pre-processing prior to analysis. Initially, a large foundation model known as the Recognize Anything Model (RAM) ^30^ will be employed. RAM specializes in image tagging and has been developed through extensive training on a large number of general images. This model will play a crucial role in identifying images captured by our customized wearable camera. Its function will be to detect the presence of food items within these images. Upon detection of food items, RAM will assign a ‘food tag’ to the relevant images. This tagging mechanism is both efficient and precise, ensuring that only images with clear food content are marked for inclusion. The images that receive a ‘food tag’ from RAM will then be segregated from the rest and earmarked for further analysis, forming the core dataset for the study. Meanwhile, we have also designed ‘excluding tags’ for our model, which include around 20 categories of items such as bathrooms, mobile phone screens, and PC screens, with the flexibility to add or remove items as needed. This design is intended to prevent sensitive items from appearing alongside food in the collected data. Furthermore, for the retained images, an additional layer of protection will be implemented by blurring the faces of the participants and other individuals residing with them, as well as any other visible phone and computer screens that were unintentionally missed in the previous step. This step will use YOLOv8, ^31^ a deep learning technique renowned in the field of image recognition, to prevent the inadvertent disclosure of identities and personal information. Only after this pre-processing will the images be subjected to further analysis.

Following pre-processing is the food recognition phase, where we will leverage large language models (LLMs) ^32^ capable of processing both text and images. These models have been extensively trained on vast datasets, allowing them to recognize a wide range of food types without the need for additional training or fine-tuning. Portion size estimation also plays a pivotal role in dietary assessment, and our approach is tailored to address the unique challenges associated with using a wearable camera that captures only red-greed-blue (RGB) images, without the depth information provided by stereo imaging. This brings us to the issue of scale ambiguity, which is a significant problem given that our system cannot rely on stereoscopic methods to estimate the volume of food items. To circumvent the need for users to place a reference object next to their food - which would be an inconvenience and could disrupt the natural eating environment - we are exploring the potential of leveraging large-scale AI foundation models to learn the general context of objects and their environmental surroundings in relation to food. By understanding these contextual relationships, the model can make more accurate inferences about the portion sizes of the food being consumed.

### Subjective dietary assessment tools

24-hour dietary recall

Participants will complete a self-administered 24-hour dietary recall following each study day to measure both the accuracy of self-reporting and usability of repeated 24-hour dietary recalls. Participants will use Intake24 ( intake24.com), which is a validated, web-based, open-source computerised dietary recall system based on the multiple pass method. ^33^ The tool, currently maintained by the University of Cambridge, Monash University and Newcastle University, is used by the UK’s National Diet and Nutrition Survey Rolling Programme. ^25, 33^ Participants will use Intake24 to record everything they ate and drank the day before from midnight to midnight by using free text to list each food/drink item consumed per meal as part of the initial ‘quick list’. Next, the detailed pass stage involves searching Intake24’s food database for the closest match for each item then estimating their portion size using the images presented on the screen. Intake24 will also prompt the participant about foods usually consumed together, e.g., if they recorded coffee then it will ask if they added milk and/or sugar, as well as frequently forgotten foods, such as condiments. Additional questions will be presented if Intake24 identifies long time gaps without food or very low energy intakes, and the recall ends with the participant reviewing their entries. Prior to completing Intake24 for the first time, participants will be encouraged to watch the tutorial video (accessible via the Intake24 menu). At the end of the study, data will be exported from Intake24 including the quantities and nutritional intakes for each food item recorded per recall, after which mean daily intakes per day per participant will be calculated.

FFQ

The eNutri web app, developed by researchers from the University of Reading, includes an FFQ based on UK diets. ^34^ The current version includes 157 food, drink and supplement items. For each food and drink item, users first select how often they consumed it during the previous 4 weeks from 10 frequency buttons (such as ‘not in the last 4 weeks’ and ‘once a day’). If consumed, they then select their typical portion size from 7 portion size photos/buttons. Certain items (n=37) also request additional details, for example, the type of milk (if any) added to their coffee and whether the item consumed was a low-fat or low-sugar variety (e.g., soft drinks, yogurts); each of these items also has an ‘I’m not sure’ option. Users also report frequency of use of salt (added at the table and/or during cooking) and 8 dietary supplements. Using this information, eNutri automatically calculates mean daily intakes (g/d) of each food item, from which it estimates a large range of food group intakes (e.g., vegetables, dairy) and nutrient intakes (e.g., protein, vitamin C). In addition to dietary intake, eNutri also records certain demographic and lifestyle information about the users (such as age, sex, ethnicity, education level, physical activity levels and smoking status).

For this study, participants will use the eNutri FFQ tool on day 1 of study period 1 to measure their habitual diet by recording what they ate and drank during the previous 4 weeks. Prior to using eNutri for the first time, participants will watch the short tutorial video on the web app. To measure dietary intake during the two 4-day study periods, a separate version of eNutri was created that adapted the frequency options to reflect 4 days of dietary intake (such as ‘not in the last 4 days’ and ‘1x in the past 4 days’).

### Participant feedback and usability of technologies

Participants will complete the system usability scale (SUS) questionnaire following their first use of eNutri (day 1, week 1) and Intake24 (day 2, week1) via REDCap. The SUS questionnaire is widely-used to “measure people’s subjective perceptions of the usability of a system” and comprises of 10 alternating positive and negative statements regarding the user experience ( Table 4). ^35^ For each statement, respondents rate their agreement on a 5-response-scale from “Strongly Disagree” to “Strongly Agree” and, using the method described by Brooke (1995), a SUS score ranging from 0 to 100 is calculated, with higher scores indicating better usability. ^36^ Overall usability is also evaluated with a general question: “Overall, I would rate the user-friendliness of this system as:” with 7 options from “Worst Imaginable” to “Best Imaginable”.

**Table 4.** SUS * questionnaire.

1	I think that I would like to use this system frequently.
2	I found the system unnecessarily complex.
3	I thought the system was easy to use.
4	I think that I would need the support of a technical person to be able to use this system.
5	I found the various functions in this system were well integrated.
6	I thought there was too much inconsistency in this system.
7	I would imagine that most people would learn to use this system very quickly.
8	I found the system very awkward to use.
9	I felt very confident using the system.
10	I needed to learn a lot of things before I could get going with this system.

* System usability scale.

At the end of the study (day 4, week 2), participants will also provide feedback about all of the tools used during the study. This includes free text and Likert questions and will be completed via REDCap.

### Statistical analysis

Previous studies have shown that the misreporting rate of total energy expenditure between self-reported dietary assessment tools and double labelled water was 35 % with a standard deviation of 33 %. ^37^ To reduce the misreporting rate to <10%, an *a priori* sample size calculation determined that to achieve a power of 80 % with a type 1 error of 5%, a total of 27 participants are required. This was increased to 30 participants to account for potential dropouts. Two separate clinical research centres will be used, with both centres recruiting participants to ensure consistent demographics between sites (sex, age, and BMI).

Bootstrapped Lin’s concordance correlation coefficient (CCC) with 95% confidence intervals will be used to test the extent of agreement between each dietary assessment tool/technology and the nutrient composition of known diets and recorded compliance.

### Primary and secondary outcomes to be measured

The primary outcome will be the accuracy of dietary reporting, measured at the end of each intervention week using dietary data collected from wearable cameras, spot urine samples, capillary blood samples, and self-reported dietary assessments.

Secondary outcome measures include: 1) the creation of a multiplatform model of dietary intake using g/day measured from wearable cameras and self-report dietary assessments, and μg/ml of dietary exposure biomarkers from spot urine samples and capillary blood samples at the end of the study, and 2) the design of a dietary intake study in a free-living population that will be informed by the results of the current dietary intake study protocol.

### Dissemination

The results of this study will be presented at medical meetings, research conferences and published open access in peer-reviewed scientific journals and lay publications, approximately six months following the end of the study. They will also be used by research students who are associated with this project in work that will contribute to their degree (BSc, MSc, PhD) or other qualification, and shared with the press and media. The datasets will be made available and deposited in public databases at the point of publication. All data will be released within 2 years of the project’s completion and will be made accessible.

## Discussion

This study provides a novel approach to dietary assessment by addressing the significant limitations present in traditional methods, such as those used in the National Diet and Nutrition Survey ^10^ and other population surveys. ^33^ The novel methods to be tested in SODIAT-1 will reduce participant burden, requiring less detailed recording of foods and drinks consumed. For individuals with conditions affecting memory, this approach also eliminates reliance on recall, thus reducing the risk of inaccurate data collection. ^13^ Furthermore, the methodology will particularly be helpful to capture dietary intake of underrepresented populations, including individuals with illiteracy, language and cultural differences as well as marginalised groups such as homeless people, whose dietary intake is difficult to capture accurately through traditional self-reporting methods and are often left out from the nationwide studies. ^38^ The ability to access these populations broadens the scope and applicability of dietary assessments, providing a more comprehensive understanding of dietary patterns across different demographics.

Despite these strengths, there are also limitations to this this approach when used in less controlled conditions. For instance, participants will be asked to keep their urine samples refrigerated before returning them to the study centre, but failure to comply could affect biomarker detection during analysis, potentially compromising the accuracy of the data. ^20^ The use of cameras to record dietary intake also presents challenges. For example, accurately measuring foods and drinks consumed directly from their packaging (e.g., cans of fizzy drinks and packets of crisps) remains difficult and amount of the leftovers cannot be detected. Additionally, distinguishing between types of foods and drinks (e.g., low-fat versus whole yoghurts or sugar-free versus sugar-sweetened drinks) through visual means can be challenging. Moreover, this method relies on participants consistently wearing and correctly using the cameras, which may not always be practical or adhered to. ^19^

### Ethical approval

The study received a favourable opinion for conduct by the Camden & Kings Cross Research Ethics Committee (23/LO/0437) on 4 ^th^ July, 2023 and the University of Reading Research Ethics Committee (23/19) on 19 ^th^ May, 2023. The study will be conducted according to the principles expressed in the Declaration of Helsinki.

### Ethical considerations

Prior to screening, potential participants will receive an ethically approved participant information sheet containing full details of the study. They will have adequate time to consider taking part in the study and have an opportunity to ask questions before attending the pre-study visit where they will give written informed consent.

When participants wear the cameras, they will collect images of the participant, people around them and their devices (e.g. smartphones). To ensure everyone’s anonymity and privacy, any people and device screens recorded on the images will be automatically blurred prior to analysis, as described above. In addition, any non-food related images will be removed from the dataset. Both processing steps will be achieved via an artificial intelligence methodology and only the pre-processed dataset will be analysed by the research team. Participants will also be advised to remove their camera when it is not appropriate to wear them (such as when in the bathroom and dressing) and to log these instances.

### Data management

The data collected through the SoDiat-1 study will be pseudonymised and anonymised. Pseudonymised data will be shared among research partners for data analysis purposes. The confidentiality of study participants will be preserved under the Data Protection Act. Acellular urine samples will be transferred to Aberystwyth university and dried blood samples will be sent to the University of Cambridge in compliance with the Human Tissue Authority (HTA) regulation. The data generated from the wearable cameras will be stored on encrypted hard drives and transferred to Imperial College London after the data collection is completed. For subjective dietary assessment tools eNutri and Intake24, participants will use pre-generated weblinks and/or login details to avoid using personal information such as email addresses and names. Other data will be input on REDCap by study researchers, which will be double checked by the study coordinator at each site before the records are locked.

### Study status

Recruitment for this study concluded in May 2024, with data collection completed in mid-June 2024. At the time of paper submission, blood and urine samples, as well as camera images, have been transferred to the respective research teams, and data analysis is currently in progress.

### Trial registration

The study was registered at ISRCTN ( ISRCTN13562899).

## Data Availability

No data is associated with this article. Zenodo: A-dual-site-dietary-intervention-study-to-integrate-dietary-assessment-methods. https://zenodo.org/records/13360114 This project contains the following extended data:
•
Dual_site_dietary_intervention_Menus.pdf (Study Meal Plans) •
Dual_site_dietary_intervention_PIS.pdf (Participant Information Sheet) • Protocol_Version1.0_15032023.pdf (Study Protocol) • Consent form • SPIRIT 2013 Checklist Data are available under the terms of the Creative Commons Zero v1.0 Universal License (CC0).

## Software availability statement

• Source code for The Recognize Anything Model (RAM) is available from: https://github.com/xinyu1205/recognize-anything (the GitHub repository)
• Source code for YOLOv8 is available from: https://github.com/ultralytics/ultralytics (the GitHub repository)
• The large language model (LLM) mentioned in this study is being developed in-house by the research team at Imperial College London and will be shared in a separate paper once completed.
