# Supplementary material for: Standardised and Objective Dietary Intake Assessment Tool (SODIAT): Protocol of a dual-site dietary intervention study to integrate dietary assessment methods
Source: F1000Res. 2025 Mar 31;13:1144. Originally published 2024 Oct 7. [Version 2] doi: 10.12688/f1000research.155683.2 (PMC12498317; doi:10.12688/f1000research.155683.2)
Supplement: Peer review history

# Reviewer report: Reviewer response for version 2

**DOI:** 10.5256/f1000research.179575.r374529

**Published:** 2025-05-13

**Authors:** Cătălina Cuparencu

**Affiliations:** 1 University of Copenhagen, Copenhagen, Denmark

I would like to thank the authors for thoroughly considering my feedback. I find that the argumentation and changes included in the text are satisfactory. The paper is a lot clearer now.

 Only a minor comment, related to the secondary outcomes. It would help the reader to get an impression of the quantitative biomarkers the authors plan to measure without needing to read the reference by Beckmann et al. (if possible to include a few examples). Best of luck to the authors with this important research!

Is the study design appropriate for the research question?

Yes

Is the rationale for, and objectives of, the study clearly described?

Yes

Are sufficient details of the methods provided to allow replication by others?

Yes

Are the datasets clearly presented in a useable and accessible format?

Not applicable

Reviewer Expertise:

biomarkers of food intake, dietary assessment, nutrition research, metabolomics

I confirm that I have read this submission and believe that I have an appropriate level of expertise to confirm that it is of an acceptable scientific standard.

# Reviewer report: Reviewer response for version 2

**DOI:** 10.5256/f1000research.179575.r377257

**Published:** 2025-05-02

**Authors:** Hidemi Takimoto

**Affiliations:** 1 National Institutes of Biomedical Innovation, Osaka, Japan

The study protocols presented are informative, and useful for researchers conducting similar research. One thing I am interested is the feasibility of wearing cameras during the study period. I wonder if it wasn't annoying for the participants to start wearing the cameras the following morning before returning to the research unit. There are no descriptions regarding physical activity status of these subjects, however, they should be addressed in future studies.

Is the study design appropriate for the research question?

Yes

Is the rationale for, and objectives of, the study clearly described?

Yes

Are sufficient details of the methods provided to allow replication by others?

Yes

Are the datasets clearly presented in a useable and accessible format?

Not applicable

Reviewer Expertise:

Nutritional epidemiology and dietary assessment methodologies.

I confirm that I have read this submission and believe that I have an appropriate level of expertise to confirm that it is of an acceptable scientific standard.

# Reviewer report: Reviewer response for version 1

**DOI:** 10.5256/f1000research.170884.r359796

**Published:** 2025-02-03

**Authors:** Emily B. Hill

**Affiliations:** 1 Department of Pediatrics, Section of Nutrition, University of Colorado Anschutz, Anschutz, Colorado, USA

**OVERVIEW AND GENERAL COMMENTS**

 This protocol describes a controlled feeding study designed to assess the effectiveness of three technologies (urine biomarkers, capillary blood biomarkers, wearable camera technology) alongside two self-report dietary assessment tools. While this is a well-defined population and study design, it is unclear if the proposed correlational analyses will be sufficient to determine “effectiveness” of these tools. Generally, this is a well-written protocol to address a need in dietary assessment that is highly relevant. It is acceptable for indexing given the general concern above and below comments are addressed.

**SPECIFIC COMMENTS**

**Abstract:** Which of the assessment methods are considered novel? Diet records and food frequency questionnaires are commonplace in nutrition research, and details regarding biomarkers and wearable technology are quite limited in the description provided. Further, is “actual” food intake (i.e., weighed/portioned food delivered in a controlled setting) the comparator for each of the five proposed assessment methods?

**Abstract:** It seems a food frequency questionnaire would be an odd choice for assessment of a four-day diet, as the purpose of this instrument is to assess usual intake over a defined period of time (typically 30 days or more). Has the eNutri tool been validated for this recall period? How will data be integrated between diet assessment instruments?

**Abstract: **Which urine and capillary biomarkers will be measured? How will they be measured (e.g., targeted vs untargeted approaches)?

**Abstract:** Authors do not describe how this study will reduce misreporting or enhance inclusivity.

**Introduction:** While a good point, stating that shorter recording periods for dietary assessment are problematic for capturing day-to-day variability seems to be counterproductive to the main rationale for conducting a study with four-day controlled feeding periods that may not be reflective of usual intake.

**Introduction: **Consider person-first language; “obese individuals” could be re-written as “individuals with obesity.”

**Methods, Study population: **What is the rationale for the BMI I/E criterion of 20-30 kg/m ^2^? This does not fully capture the “normal” range (18.5-24.9 kg/m ^2^) and does include those with obesity (BMI³30 kg/m ^2^). Thus, this range seems a bit arbitrary.

**Methods, Study diets:** Were diets designed only to be matched in terms of nutrient composition, or were similar foods/food groups balanced as well? Authors state “foods and drinks for each diet were selected to allow investigation of specific biomarkers,” but this does not shed much light on the details for these selections.

**Methods, Study diets:** It remains unclear which specific biomarkers will be employed/tested, though references are provided under the “spot urine samples” and “capillary blood samples” sections. It may be helpful to include examples of these compounds in table format. Is a one-week washout period adequate based on the expected half-life of these biomarkers, and are they expected to be sufficiently different between diets?

**Methods, Study visit procedures/24-hour dietary recall/FFQ:** It appears habitual diet as assessed on Day 1 of the protocol using the eNutri tool reflects usual intake prior to the study period? How is it used in this study? Why is Intake24 not used on Day 1?

**Methods, Objective dietary assessment tools:** Information on the type of analysis (metabolomics at minimum) would be helpful to include earlier in the protocol.

**Methods, Objective dietary assessment tools/Capillary blood samples:** Is a capillary blood draw completed only in the morning of each study visit on Days 1, 2, and 4? What is the rationale for excluding day 3?

**Methods, Wearable camera technology:** It is not clear why the participants will be asked to continuously wear the mounted camera outside of eating windows.

**Methods, FFQ:** As mentioned in the abstract, it is not clear that the eNutri tool has been validated for four-day recall periods – is part of the objective of this study to validate the “separate version” that was created? If so, how will this be done?

**Methods, Statistical analysis:** Are simple measures of correlation between instruments adequate to measure accuracy? Are the biomarkers selected indicative of specific nutrients (e.g., fat, sodium), foods, or food groups? If the latter are not controlled for or not substantially different between diets, what is the expectation for how accuracy will be determined if the biomarkers do not distinguish at this level? Where will participants report/record compliance? Or is the study team measuring this? Details are lacking for evaluation of these methods.

**Methods, Primary outcomes:** How will “accuracy” be determined amongst the data collected? Which measure is “true?”

**Methods, Secondary outcomes:** It is not clear how this multiplatform model will be designed or how all of the assessment measures will be integrated together. Further, with no detail on the biomarkers of interest, it is impossible to evaluate if the goal of assessing ug/mL of dietary exposure biomarkers is realistic. Many untargeted metabolomics assays do not provide data at this level (e.g., relative abundance vs quantitative measures/concentrations). While the use of triple quad indicates some targeted panels may be run and thus this may be possible, the stated goal to create a simple “global dietary score” from NMR data does not align with this stated objective.

**Discussion:** While the end goal is to reduce participant burden, this reviewer would argue the study increases it given the number of data collection procedures. Consider specifying that if the methods tested are reliable, reproducible, etc. and can be incorporated into clinical practice and/or future research, this could reduce burden.

**Discussion:** Similar to comments in the abstract, it is not immediately clear how these methods will improve assessment in underrepresented populations, particularly those who are experiencing homelessness, as stated in text. It would be near impossible to give someone the wearable technology or ask them to appropriately collect and store biospecimens such as urine under those conditions.

**Discussion:** Consider adding to strengths/limitations the length of collection for blood (small capillary sample vs venous draw) and urine (spot vs 24 hour). While these methods may reduce participant burden, they also weaken the ability to capture precise measures reflective of longer periods of intake.

**Ethical considerations:** While perhaps outside the scope of this protocol manuscript, it is not clear how AI technology being employed is protective of personally identifiable information – what program “sees” this? The reviewer notes the software availability statement but feels this may be inadequate.

Is the study design appropriate for the research question?

Partly

Is the rationale for, and objectives of, the study clearly described?

Yes

Are sufficient details of the methods provided to allow replication by others?

Partly

Are the datasets clearly presented in a useable and accessible format?

Not applicable

Reviewer Expertise:

dietary assessment, metabolomics, nutrition intervention, behavior change

I confirm that I have read this submission and believe that I have an appropriate level of expertise to confirm that it is of an acceptable scientific standard, however I have significant reservations, as outlined above.

# Response

**Published:** 2025-03-20

**Authors:** Eka Bobokhidze

**Affiliations:** Food and Nutritional Sciences, University of Reading School of Chemistry Food and Pharmacy, Reading, England, UK

Dear Emily

 Thank you for your feedback and comments on our manuscript. We have refined the relevant sections to reflect your thoughtful review, and we appreciate the opportunity to strengthen our work.

 Yours Sincerely,

 Julie A. Lovegrove

 On behalf of the manuscript authors

Response to the reviewer's comments:

**1. Abstract: Which of the assessment methods are considered novel? Diet records and food frequency questionnaires are commonplace in nutrition research, and details regarding biomarkers and wearable technology are quite limited in the description provided. Further, is “actual” food intake (i.e., weighed/portioned food delivered in a controlled setting) the comparator for each of the five proposed assessment methods?**

 The tools used are combination of both well-established methods and emerging technologies. While each individual tool may not be considered novel on its own, as the tools used are combination of both well-established methods and emerging technologies, the unique combination of these tools together and their integration of tools to improve reporting accuracy is, can be considered a novel approach. Due to the abstract word limit (300 words) this information has been included at the end of the Introduction (line 52-54).

 The comparator for each tool will be the known food intake and corresponding nutrient intake of the two recorded diets that were consumed by all participants in the controlled environment. 

**2. Abstract: It seems a food frequency questionnaire would be an odd choice for assessment of a four-day diet, as the purpose of this instrument is to assess usual intake over a defined period of time (typically 30 days or more). Has the eNutri tool been validated for this recall period? How will data be integrated between diet assessment instruments?**

 In SODIAT-1, we utilised two versions of eNutri: a 4-week version to assess habitual intake and a 4-day version to specifically capture participants' food consumption during both study periods. Although FFQs typically report intakes over a longer period, they can also be used for shorter timeframes. As identified by the DIET@NET consortium “FFQs ranged from the previous day to usual intake over the previous year with 11 (32%) measuring long-term intake (>6 months) and six (16%) measuring short-term intake (one day)” ( Hooson et al, 2019).

 The data from the 4-day FFQ will provide an average intake across the two study periods, allowing integration with other daily aggregated data, such as Intake24. This information will be analysed alongside metabolomic data and images to evaluate consistency and identify potential biases. Its inclusion is essential for assessing the alignment between self-reported dietary data and objective biomarkers, directly contributing to the trial’s overarching goal of improving accuracy in dietary assessment methods. We added more details about eNutri data integration in the statistical analysis section (line 355-361).

 The validation of eNutri (4-day FFQ) against the known 4-day dietary intake data from each controlled diet will be evaluated after the formal study analysis.

**3. Abstract: Which urine and capillary biomarkers will be measured? How will they be measured (e.g., targeted vs untargeted approaches)?**

 Authors will publish the detailed list of biomarkers alongside the results as such information is outside of the scope of protocol paper. The analysis of known intake biomarkers in urine and blood is with targeted quadrupole mass spectrometry methods for reporting of primary and secondary outcomes. Due to the word count limitation this information was not included in the abstract and can be found in the sub-section of “Spot urine samples” (line 199-205).

**4. Abstract: Authors do not describe how this study will reduce misreporting or enhance inclusivity.**

 In response to the both reviewers’ comments, we have revised this section to clarify that the overarching goal of reducing misreporting and increasing inclusivity applies to the broader SODIAT project, rather than specifically to the SODIAT-1 study. Further details on how the SODIAT project aims to achieve this objective are provided in the Discussion section of the main text.

**5. Introduction: While a good point, stating that shorter recording periods for dietary assessment are problematic for capturing day-to-day variability seems to be counterproductive to the main rationale for conducting a study with four-day controlled feeding periods that may not be reflective of usual intake.**

 This highly controlled clinical trial was not designed to reflect participants' usual intake during the 4-day study periods. Instead, its primary objective was to calibrate each tool and technology against a known intake (controlled menus) and evaluate whether they, individually and in combination, can accurately represent food consumption. Findings from the SODIAT-1 study will inform data-driven modelling approaches for subsequent studies. In SODIAT-2, the research team plans to test these tools in a free-living population over a longer period (5 weeks).

**6. Introduction: Consider person-first language; “obese individuals” could be re-written as “individuals with obesity.”**

 The authors appreciate this suggestion and the appropriate amendments were maid in the text (line 32).

**7. Methods, Study population: What is the rationale for the BMI I/E criterion of 20-30 kg/m ^2^? This does not fully capture the “normal” range (18.5-24.9 kg/m ^2^) and does include those with obesity (BMI³30 kg/m ^2^). Thus, this range seems a bit arbitrary.**

 The sample size calculation to ensure that this study was adequately powered was based on data from the Pettitt et al 2016, on the mis-reporting rate of total energy expenditure. While the BMI range we used for our inclusion criteria is not considered the “normal” range, it reflects the BMI range of participants from the Pettitt et al 2016 study. Additionally, as the mean BMI of UK adults is 27.6 Kg/m 2, a BMI range of 20-30 Kg/m2 is more representative of the UK population.

**8. Methods, Study diets: Were diets designed only to be matched in terms of nutrient composition, or were similar foods/food groups balanced as well? Authors state “foods and drinks for each diet were selected to allow investigation of specific biomarkers,” but this does not shed much light on the details for these selections.**

 Two (4 day) isoenergetic diets were designed one of which had a nutritional portfolio that complied to current UK healthy eating guidelines and another that related to an unhealthy dietary profile. We have demonstrated that this methodology produces distinct metabolomic profiles (DOI: 10.1016/S2213-8587(16)30419-3). Each of the diets were made up from a variety of commonly consumed foods taken from the NDNS dietary profiles of people who consume a healthy and unhealthy dietary profile. All foods and drinks offered to the volunteers were weighed as was as all unconsumed foods and drinks. This tested the research tools’ ability to pick up dietary differences.

**9. Methods, Study diets: It remains unclear which specific biomarkers will be employed/tested, though references are provided under the “spot urine samples” and “capillary blood samples” sections. It may be helpful to include examples of these compounds in table format. Is a one-week washout period adequate based on the expected half-life of these biomarkers, and are they expected to be sufficiently different between diets?**

 While we welcome the Reviewers suggestion, the authors feel that detailed reporting of biomarker lists and detailed discussions on wash-out periods, half-life and their utility, etc... would be better placed within a publication of the trial results; not in a trial protocol paper. The biomarkers selected have been validated in controlled intervention and free-living experiments to ensure that they are representative of foods commonly consumed in UK.

**10. Methods, Study visit procedures/24-hour dietary recall/FFQ: It appears habitual diet as assessed on Day 1 of the protocol using the eNutri tool reflects usual intake prior to the study period? How is it used in this study? Why is Intake24 not used on Day 1?**

 The FFQ data collected at baseline will be used to assess habitual diet for the purpose of reporting cohort demographics and may be used as a covariate in data analysis to control for differences in habitual diet between participants. We added this information in Statistical analysis section (Line 356-357).

 Participants completed Intake24 (a self-administered 24-hour dietary recall) to records their dietary intake during the study periods. To measure both the accuracy of self-reporting and usability of repeated 24-hour dietary recalls, participants were asked to complete Intake24 after each study day and recall their intake during the previous day. As there was nothing to recall on day 1, participants started using Intake24 on day 2 of each study week.

**11. Methods, Objective dietary assessment tools: Information on the type of analysis (metabolomics at minimum) would be helpful to include earlier in the protocol.**

 These methods have been published in detail elsewhere and therefore authors think it is outside of the scope of the manuscript (DOI:10.1002/mnfr.202000517).

**12. Methods, Objective dietary assessment tools/Capillary blood samples: Is a capillary blood draw completed only in the morning of each study visit on Days 1, 2, and 4? What is the rationale for excluding day 3?**

 The first sample (day 1) was a test sample for the participant to become familiar with the Onedraw device. The key samples are on day 2 and day 4. Taking a sample on day 3 would not service a specific purpose and therefore not be ethical to take unnecessary blood sample and create a significant increase in the costs of the work.

**13. Methods, Wearable camera technology: It is not clear why the participants will be asked to continuously wear the mounted camera outside of eating windows.**

 The SODIAT project aims to develop a tool that is practical and works in real-life settings. While SODIAT-1 was a highly controlled study and not fully representative of a real-world environment, our goal was to evaluate the feasibility of using camera technology throughout most of the day, both inside and outside the clinical unit. We were concerned that if participants were only required to wear the cameras during eating windows, they might forget to put them on before consuming food or beverages in a real-life setting, particularly for spontaneous eating events such as snacks and drinks.

**14. Methods, FFQ: As mentioned in the abstract, it is not clear that the eNutri tool has been validated for four-day recall periods – is part of the objective of this study to validate the “separate version” that was created? If so, how will this be done?**

 While the validation of the 4-day eNutri FFQ is not a primary focus of the SODIAT project, the authors have considered this possibility. Specifically, we have explored the potential for validating eNutri against the known 4-day intake from the controlled menus and, possibly, against dietary data collected through Intake24.

**15. Methods, Statistical analysis: Are simple measures of correlation between instruments adequate to measure accuracy? Are the biomarkers selected indicative of specific nutrients (e.g., fat, sodium), foods, or food groups? If the latter are not controlled for or not substantially different between diets, what is the expectation for how accuracy will be determined if the biomarkers do not distinguish at this level? Where will participants report/record compliance? Or is the study team measuring this? Details are lacking for evaluation of these methods.**

****

 To clarify, accuracy will not be measured between instruments. The primary reporting accuracy will be measured between each tool and the known intake (as clarified in statement 1).

 Most dietary components will be metabolised and will not be identical to their originating food components. For instance, palmitate will be in food mainly as an ester part of the triglycerides (oils), but in the circulation it can be part of phospholipids, different triglycerides or other lipids and therefore the measurement of those lipids in the circulation is not directly related to the intake. However, measuring the absolute concentrations will allow for direct comparison with other studies, while relative concentrations are very dependent on all the other compounds measured at the same time. So, the quantitation is aimed to make the results as transferable as possible. The imprecision of the sampling is an issue and will always be a limitation, but quantitation of the biomarkers in that particular sample will always be more transferable than relative amounts, which can always be calculated from the absolute amounts. 

 Compliance was recorded by study investigators when the participants were in the controlled environment, and for the time outside the controlled environment compliance was measured using sample/data collection records and self-reported deviations to study menus (added in the main text line 182-185).

**16. Methods, Primary outcomes: How will “accuracy” be determined amongst the data collected? Which measure is “true?”**

 The true measure will be the known quantities of consumed foods given to participants during the intervention days within the clinical trial centres. We clarified this in the manuscript (line 368-369). Accuracy will be measured at the level relative to the dietary assessment tool. For example, Intake24 reports nutrient level data, where biomarkers report at the food group level.

**17. Methods, Secondary outcomes: It is not clear how this multiplatform model will be designed or how all of the assessment measures will be integrated together. Further, with no detail on the biomarkers of interest, it is impossible to evaluate if the goal of assessing ug/mL of dietary exposure biomarkers is realistic. Many untargeted metabolomics assays do not provide data at this level (e.g., relative abundance vs quantitative measures/concentrations). While the use of triple quad indicates some targeted panels may be run and thus this may be possible, the stated goal to create a simple “global dietary score” from NMR data does not align with this stated objective.**

 The analysis of known intake biomarkers in urine and blood is with targeted quadrupole mass spectrometry methods. For the majority of the biomarkers, the inclusion of authenticated standards allows for the quantitative measurements. For some biomarkers where standards are unavailable, quantification is performed using a surrogate, with similar physiochemical properties and ionisation behaviour; within the specific sample matrix. This will mean that some of the concentrations from targeted analyses, will be semi-quantitative.  

 The secondary analysis using NMR to create a global dietary score, is based on a previously validated methods (DOI: 10.1016/S2213-8587(16)30419-3), and provides complementation to mass spectrometry analysis.  

**18. Discussion: While the end goal is to reduce participant burden, this reviewer would argue the study increases it given the number of data collection procedures. Consider specifying that if the methods tested are reliable, reproducible, etc. and can be incorporated into clinical practice and/or future research, this could reduce burden.**

 While we acknowledge the reviewer’s comment, we would like to emphasise that reducing participant burden is the overarching goal of the SODIAT project. We recognise that the SODIAT-1 study, due to its extensive data collection procedures, is not the best example of this aim. However, we hope the clarifications made in the Discussion section help to better convey this point. Additionally, the findings from SODIAT-1 will inform researchers in identifying the most effective combinations of study tools, allowing future studies to be less burdensome for participants while not undermining the date quality. Ultimately, insights from the SODIAT-2 study will contribute to developing a more streamlined and inclusive research tool that minimises participant burden by reducing the need for recall or requiring minimal input (for example, shorter FFQ that adds on the biomarker and camera data).

**19. Discussion: Similar to comments in the abstract, it is not immediately clear how these methods will improve assessment in underrepresented populations, particularly those who are experiencing homelessness, as stated in text. It would be near impossible to give someone the wearable technology or ask them to appropriately collect and store biospecimens such as urine under those conditions.**

 We appreciate the reviewer’s comment and acknowledge the challenges of conducting dietary research among individuals experiencing homelessness. In the remote SODIAT-2 study, participants will collect their urine and blood samples at home and mail them to research centres using pre-paid envelopes. We believe this approach can be adapted to accommodate the circumstances of individuals experiencing homelessness, although we recognise it presents additional challenges. Ultimately, the final SODIAT tool aims to address these difficulties and improve dietary assessment methodologies for underrepresented populations, including those without stable housing.

**20. Discussion: Consider adding to strengths/limitations the length of collection for blood (small capillary sample vs venous draw) and urine (spot vs 24 hour). While these methods may reduce participant burden, they also weaken the ability to capture precise measures reflective of longer periods of intake.**

 We think that it is too early to give a detailed strengths/limitations discussion on the length of sample collection. We have no evidence at the moment that for the measurement of the biomarkers in 24h urine or venous blood samples are more informative than the measurement in spot urine or capillary blood samples. Neither methods are functional as quantitative biomarkers (like the sodium measured in 24h urine samples can be used to calculate salt consumption). The biomarkers are likely to be used as qualitative biomarkers, revealing consumption and change of consumption of specific food or food groups. Very few projects have integrated blood based and urine based biomarkers and our work will enable the further development of these biomarkers in the integrated projects.

**21. Ethical considerations: While perhaps outside the scope of this protocol manuscript, it is not clear how AI technology being employed is protective of personally identifiable information – what program “sees” this? The reviewer notes the software availability statement but feels this may be inadequate.**

 The “recognise anything” model automatically tags each captured image, ensuring that only food-related images are retained for further analysis. Our experiments demonstrate that more than 99.5% of redundant images are removed, significantly reducing the risk of handling non-relevant data.

 To further protect personally identifiable and sensitive information such as faces, laptop and phone screens, documents, we apply blurring using YOLOv8, a state-of-the-art (SOTA) deep learning model for image recognition. This process achieves an accuracy rate of 99.8%, effectively safeguarding participants' identities and those around them, also blurring screens and paper documents if captured by the camera technology.

 Regarding software availability, we are currently working on a research article that will provide detailed information about the models used, as the anonymisation technique itself represents a novel aspect of this project. The specifics of our approach will be disclosed in that publication.

# Reviewer report: Reviewer response for version 1

**DOI:** 10.5256/f1000research.170884.r341524

**Published:** 2024-12-17

**Authors:** Cătălina Cuparencu

**Affiliations:** 1 University of Copenhagen, Copenhagen, Denmark

**Comments:**

 The manuscript describes the protocol for a study designed to integrate dietary assessment tools covering traditional technologies (FFQs and recalls) with last-generation technologies (biomarkers of food intake measured in biological samples and wearables). The study is great in many ways and has the potential to shed light into the applicability and cost-effectives of the new dietary assessment tools, as well as providing a first attempt to cross-validate these tools against each other. The concept is novel and highly needed to move dietary assessment forward in nutrition research and provide a more accurate estimate of the human diet.

 Some considerations:

**Abstract:**

 The cross-over design is 2x4 days with wash out. How will the FFQs information be integrated with the acute dietary information?

 Dietary intake will be monitored continuously through wearable cameras (abstract). I would imagine the cameras (glasses) have to be taken off at times (also stated by the authors later in the abstract)? Maybe “continuously” is not the best term here.

 In the discussion point (abstract) it is unclear how the study will enhance the inclusivity of underrepresented populations with literacy or language barriers, since the study will primarily recruit from Imperial College London and Reading University. If this is a perspective (future application of this methodology / impact), it should be seeded in the introduction, for coherence.  

 I suggest the authors be somewhat more cautious when stating “reducing misreporting and enhancing inclusivity”. The methodologies they will test are far from being validated and fully accurate, and the cross-validation still relies on participants turning the wearables on. It is therefore yet to be investigated if this integrative approach will indeed reduce misreporting.

**Introduction -** I suggest cautiousness in classifying wearables as part of “objective” measures, since they essentially also rely on the will of the study population to actually record everything.

**Recruitment **– it is stated that recruitment will start in December 2023 -> change to started? although later in the manuscript it is stated that it is also already completed. This can be already stated from the start.

**Study design:**

- In what cases will the washout before study not be needed? (Figure 1)
- Habitual diet will be recorded with the eNutri tool. How is this data relevant to the study?
- It is slightly unclear if day 2-4 are conducted also at the research center? I would assume not, but some clarification with help the reader

**Objective dietary assessment tools**

- How will the spot urines be normalized?
- Is the blood collection device standardized for volume of blood, or how would this be quantified?
- Wearables: very interesting technology developed, that takes into account some limitations of such technologies (eg privacy). Few points: is the recorded dietary data connected to the same food composition database as the subjective tools used (eNutri, intake24)? How does the dietary recording work with stacked foods (i.e., burgers or sandwiches) to disentangle the identity and quantities of the ingredients? What about composite meals?
- Wearables: here it is stated that participants come to the research center days 2-4; yet, later the authors refer to “natural eating environment”. This should be clarified, as an experimental diet, even if designed according to national dietary guidelines, it is by no means a natural eating environment, even more so if it is consumed at a research center

**Primary outcome:** what will be the “golden standard” among the 4 methodologies? In other words, how do the authors define “the accuracy of dietary reporting”? Logically, it would be the reflection of the diet in comparison with the dietary intervention – if this is correct, it should be clearly stated. Moreover, will this be measure at the nutrient, food group or dietary pattern level?

**Secondary outcome: **ug/ml of dietary exposure biomarker indicates quantitative biomarkers. This may be true for nutrients, but most foods are not covered by quantitative biomarkers reflecting their intake. Considering the spot sampling with potential imprecise information of volume (leading to inaccurate urines concentration, potentially also for blood spots, depending on the device used), quantifying amounts consumed with biomarkers can be challenging. What are the authors’ thoughts on this?

**Ethics:** how is the data privacy through AI being monitored?

Is the study design appropriate for the research question?

Yes

Is the rationale for, and objectives of, the study clearly described?

Yes

Are sufficient details of the methods provided to allow replication by others?

Yes

Are the datasets clearly presented in a useable and accessible format?

Not applicable

Reviewer Expertise:

biomarkers of food intake, dietary assessment, nutrition research, metabolomics

I confirm that I have read this submission and believe that I have an appropriate level of expertise to confirm that it is of an acceptable scientific standard, however I have significant reservations, as outlined above.

# Response

**Published:** 2025-01-29

**Authors:** Eka Bobokhidze

**Affiliations:** Food and Nutritional Sciences, University of Reading School of Chemistry Food and Pharmacy, Reading, England, UK

Dear Reviewer,

 We appreciate your feedback and insights on our submission to F1000Research. Below we have outlined our responses to each of the points raised during the review process.

 Yours Sincerely,

 Julie A. Lovegrove

 On behalf of the manuscript authors

 Response to the reviewer's considerations.

 1. **The cross-over design is 2x4 days with wash out. How will the FFQs information be integrated with the acute dietary information?**

 Thank you for this query. The FFQ data collected at baseline will be used to assess habitual diet for the purpose of reporting cohort demographics and may be used as a covariate in data analysis to control for differences in habitual diet between participants.

 The 4-day FFQs were then administered at the end of each study week, specifically recording what participants consumed during the previous 4 study days in which they ate the test diets. This FFQ data will provide average intakes across the  two study weeks, which can be integrated with daily aggregated data, like Intake24. This data will be compared with metabolomic data and images, enabling evaluation of consistency and identifying potential biases. Its inclusion is essential to test how well self-reported dietary data aligns with objective biomarkers, directly addressing the trial’s core aim of improving accuracy in dietary assessment methodologies.

 We clarified this in abstract and added further details in the data analysis section.

**2. Dietary intake will be monitored continuously through wearable cameras (abstract). I would imagine the cameras (glasses) have to be taken off at times (also stated by the authors later in the abstract)? Maybe “continuously” is not the best term here.**

 This is correct. Participants wore the cameras all the time while being in the research unit (until 6pm) and they were asked to continue to wear them continuously after leaving too. The glasses with the cameras were only taken off during the inappropriate times, such as using the bathroom, bathing children, while driving for safety reasons, etc. We have removed both instances of the word “continuously” from the abstract after considering the reviewer’s comment. In addition, we have removed the same word from the main text (line 237) and revised the phrasing in line 168 for clarity.

 3. **In the discussion point (abstract) it is unclear how the study will enhance the inclusivity of underrepresented populations with literacy or language barriers, since the study will primarily recruit from Imperial College London and Reading University. If this is a perspective (future application of this methodology / impact), it should be seeded in the introduction, for coherence.**

**I suggest the authors be somewhat more cautious when stating “reducing misreporting and enhancing inclusivity”. The methodologies they will test are far from being validated and fully accurate, and the cross-validation still relies on participants turning the wearables on. It is therefore yet to be investigated if this integrative approach will indeed reduce misreporting.**

 The authors note that the original text was misleading as it suggested participants were all *from* Imperial College London and the University of Reading. This has now been edited to make it clear that these were the two study locations, not the location for recruitment. Participants were recruited from the areas local to the two study sites and included people with differing demographics.

 The long-term goal of the SODIAT project is to develop a dietary intake assessment tool that will enable us to enhance inclusivity of underrepresented populations (e.g., by reducing reliance on memory recall and negating the need for high literacy skills) as well as minimise the current biases of traditional tools that contribute to misreporting. We have edited the text to clarify that these are the overall aims of the SODIAT project (rather than suggesting this will be achieved from this first study) and noted that this is the first of three studies to evaluate the novel tool.

 4. **Introduction -** **I suggest cautiousness in classifying wearables as part of “objective” measures, since they essentially also rely on the will of the study population to actually record everything.**

 Wearable cameras are considered objective measures in dietary assessment because they capture visual evidence of what participants consume without relying on self-reported data, which is prone to memory errors or biases. This is in line with how other authors outside of our research group also categorize wearable cameras – some examples include: Chan et al 2021 ( https://doi.org/10.3390/nu13061806), Scott et al 2022 ( https://doi.org/10.1017/jns.2022.81), Gemming et al 2015 (https://doi.org/10.1016/j.appet.2015.05.019).

 However, we agree that this objectivity depends on whether participants consistently wear the devices as instructed. This is noted as a potential limitation of the camera technology (line 388-389) and mentioned it in the introduction when discussing objective measures as the will of participants could also apply to the collection of biosamples (line 45). 

 5. **Recruitment** **– it is stated that recruitment will start in December 2023 -> change to started? although later in the manuscript it is stated that it is also already completed. This can be already stated from the start. (statement in rec section)**

 The paper was written while the study was in planning and early running stages, thus the wording. To avoid the confusion among the readers, we have updated the Study Status Statement and re-worded the appropriate sections to reflect that the study is now finished. We will update the sections about data analysis once its finished.

 6. **In what cases will the washout before study not be needed? (Figure 1)**

 The washout before study was applied if the eligible participant was taking any dietary supplement or participated in another study during last 12 weeks. In other cases, no washout period was needed. This detail was added to the Figure 1.

 7. **Habitual diet will be recorded with the eNutri tool. How is this data relevant to the study?**

 The FFQ data collected at baseline will be used to assess habitual diet for the purpose of reporting cohort demographics and may be used as a covariate in data analysis to control for differences in habitual diet between participants. As mentioned in the reply to the comment 1, details about how the data from habitual data will be used has been added in the statistical analysis section (line 348-354).

 8. **It is slightly unclear if day 2-4 are conducted also at the research center? I would assume not, but some clarification with help the reader**

 Participants visited and stayed in study centres from 8am to 6pm during 4 days (Monday-Thursday) and came back for a short visit on Friday morning to drop off the Friday morning urine samples and study equipment. This is described in “Study visits” and “Study visit procedures” sections.

 9. **How will the spot urines be normalized?**

 Spot urine samples are normalised based on specific gravity prior to extraction. Specific gravity correction factors are calculated for urine samples as a fold change of each urine specific gravity to a value of 1.006. This information has been added to the “Spot urine samples” section (line 196-199).

 10. **Is the blood collection device standardized for volume of blood, or how would this be quantified?**

 OneDraw blood collection kit is a standardised FDA approved device that collects approximately 150 µl of blood at each administration. This detail was added in the text (line 208).

 11. **Wearables: very interesting technology developed, that takes into account some limitations of such technologies (eg privacy). Few points: is the recorded dietary data connected to the same food composition database as the subjective tools used (eNutri, intake24)? How does the dietary recording work with stacked foods (i.e., burgers or sandwiches) to disentangle the identity and quantities of the ingredients? What about composite meals?**

 Our system is not currently integrated with the databases used by eNutri or intake24 and instead uses our own set of food categories. However, aligning our categories with intake24 is something we are actively considering, as it would improve consistency and comparability for future analyses, particularly in large-scale dietary studies.

 For stacked foods, such as burgers or sandwiches, our passive monitoring approach captures images of the internal components when the food is bitten into. This allows for more accurate identification of individual ingredients and estimation of portion sizes, even in complex food arrangements.

 When it comes to composite meals, such as curry rice, our method is designed to identify and list visible ingredients along with their estimated portions. Admittedly, this is challenging when some ingredients are covered or obscured and has been included in the limitations section. To address these complexities, we are exploring advanced solutions like leveraging large language models (LLMs), which have shown significant promise in separating ingredients and estimating portions with greater accuracy.

 To consider reviewer’s comment we have added a sentence about composite meals and stacked foods in the discussion section of the main text as follows: “Additionally, distinguishing between types of foods and drinks (e.g., low-fat versus whole yoghurts or sugar-free versus sugar-sweetened drinks) through visual means can be challenging as well as composite meals (e.g., pies, curries) and stacked foods (e.g., sandwiches, burgers) where some ingredients are covered or obscured.”

 12. **Wearables: here it is stated that participants come to the research center days 2-4; yet, later the authors refer to “natural eating environment”. This should be clarified, as an experimental diet, even if designed according to national dietary guidelines, it is by no means a natural eating environment, even more so if it is consumed at a research center.**

 We acknowledge that the participants consumed these meals in an experimental setting at the research centre, which is not representative of a natural eating environment. To clarify, the phrase in the text refers to the design method used in the study, rather than environment itself; we have amended the word ‘the’ to ‘a’ to avoid confusion.

 13. **Primary outcome: what will be the “golden standard” among the 4 methodologies? In other words, how do the authors define “the accuracy of dietary reporting”? Logically, it would be the reflection of the diet in comparison with the dietary intervention – if this is correct, it should be clearly stated. Moreover, will this be measure at the nutrient, food group or dietary pattern level?**

 The gold standard will be the known quantities of consumed foods given to participants during the intervention days within the clinical trial centres. Accuracy will be measured at the level relative to the dietary assessment tool. For example, Intake24 reports nutrient level data, where biomarkers report at the food group level.

 14. **Secondary outcome: ug/ml of dietary exposure biomarker indicates quantitative biomarkers. This may be true for nutrients, but most foods are not covered by quantitative biomarkers reflecting their intake. Considering the spot sampling with potential imprecise information of volume (leading to inaccurate urines concentration, potentially also for blood spots, depending on the device used), quantifying amounts consumed with biomarkers can be challenging. What are the authors’ thoughts on this?**

 The reviewer raises an interesting point, indeed essential nutrients exogenous compounds, can be directly correlated to intake. However most dietary components will be metabolised and will not be identical to their originating food components. For instance, palmitate will be in food mainly as an ester part of the triglycerides (oils), but in the circulation it can be part of phospholipids, different triglycerides or other lipids and therefore the measurement of those lipids in the circulation is not directly related to the intake. However, measuring the absolute concentrations will allow for direct comparison with other studies, while relative concentrations are very dependent on all the other compounds measured at the same time. So, the quantitation is aimed to make the results as transferable as possible. The imprecision of the sampling is an issue and will always be a limitation, but quantitation of the biomarkers in that particular sample will always be more transferable than relative amounts, which can always be calculated from the absolute amounts.

 In the particular case of spot urine samples, while there are limitations due to the temporal heterogeneity of urine excretion; there are well documented examples ( https://doi.org/10.1093/jn/nxz138) on the utility of spot samples in comparison to 24-hour urine samples, and the correspondence in biomarker concentrations between sample types for reporting dietary exposure.

**15. Ethics: how is the data privacy through AI being monitored?**

 All footage will undergo 2-step pre-processing procedure. Initially, RAM (described in “Software”) will be applied to remove the images that do not contain foods or drinks. Additionally, this method will be used to remove the photos with sensitive information – for example, “excluding tags” function will allow to remove pictures that contain bathroom. Additionally, an extra layer of security will be applied to the retained images by blurring participants' faces, the faces of others present, and any visible phone or computer screens that may have been overlooked in the initial step. This process will utilize YOLOv8, a highly regarded deep learning method in image recognition, to ensure that identities and personal information remain protected. Images will only proceed to further analysis after this pre-processing step. Additionally, regular audits of AI pre-processes and results will be conducted through sampling to ensure compliance and address any emerging privacy concerns throughout the experiment. We reflected this in the ethics section (line 417)
